# Supplementary material for: Genome-Wide Identification and Characterization of the bHLH Gene Family and Its Response to Abiotic Stresses in Carthamus tinctorius
Source: Plants (Basel). 2023 Nov 3;12(21):3764. doi: 10.3390/plants12213764 (PMC10648185; doi:10.3390/plants12213764)
Supplement: Supplementary file 1 [file plants-12-03764-s001.zip › plants-2637947-supplementary.pdf]

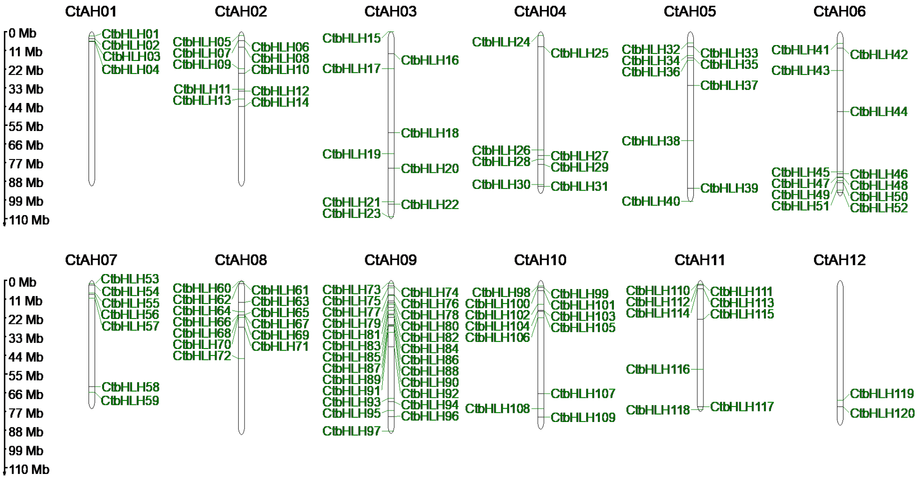

Figure S1. Chromosomal locations of the *CtbHLH* genes. The 120 *CtbHLH* genes were distributed on 12 pseudo-chromosomes of *C. tinctorius* based on their physical position.

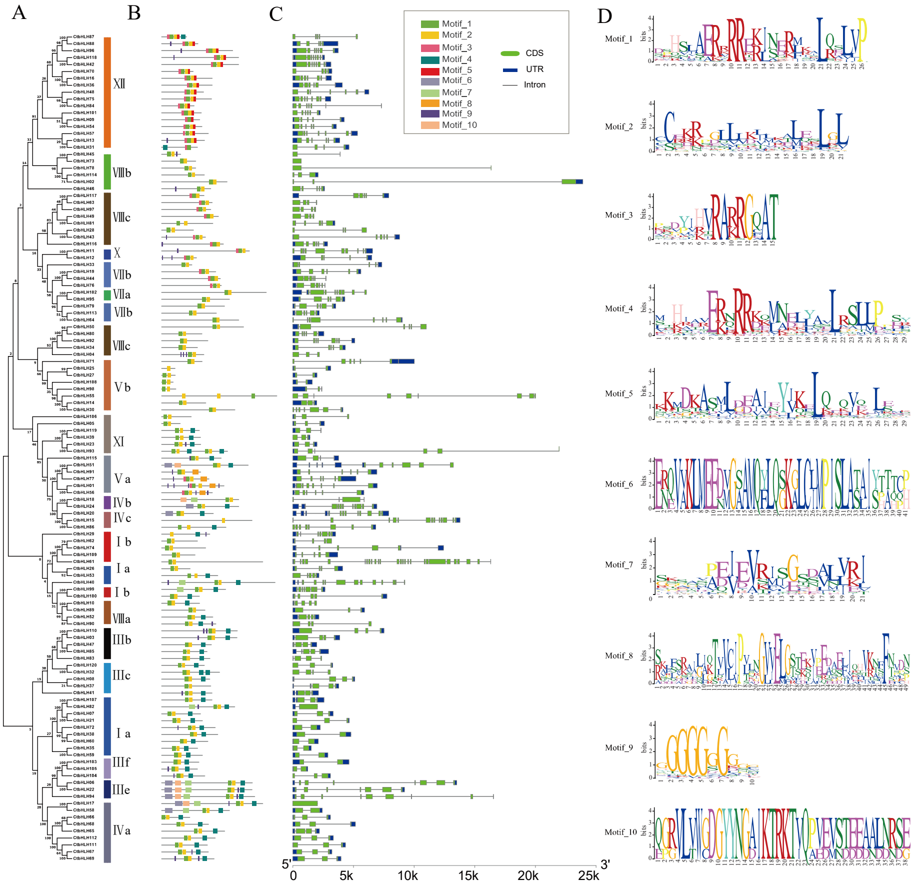

Figure S2. (A) Phylogenetic tree of CtbHLHs constructed using MEGA7.0 software with the neighbor joining method based on the alignments of complete predicted protein sequences of CtbHLH genes; (B) Conserved motif distributions of the CtbHLH genes of 120 proteins were identified using MEME-Suite 5.1.1, different colors represent different motifs; (C) Exon–intron structural analysis of CtbHLH genes

were determined using TBtools v1.130, where blue and green boxes represent untranslated regions and exons , respectively, while black lines represent introns; (D) Sequence logos for motif 1-10.

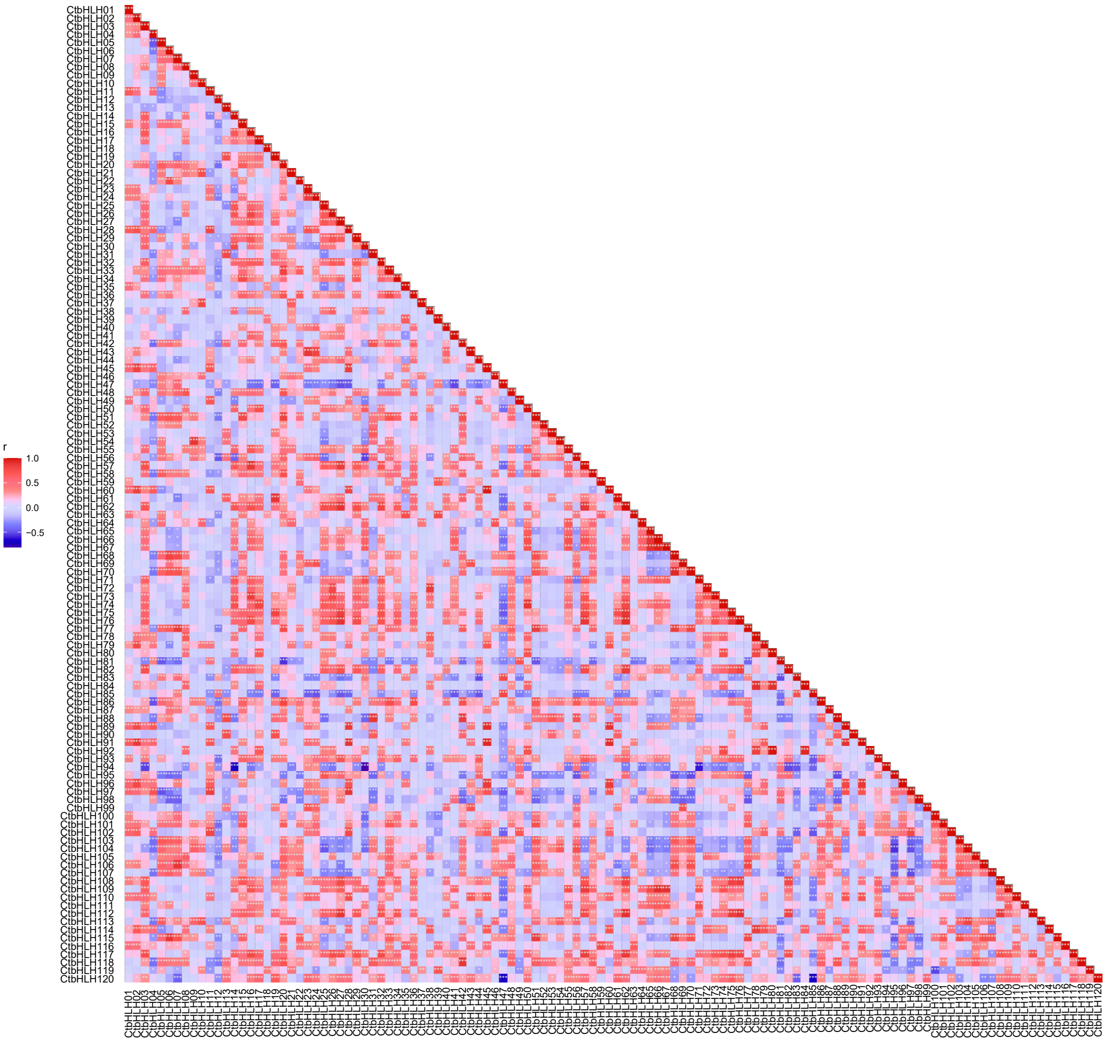

Figure S3. Co-expression analysis of *CtbHLH* genes. * $p < 0.05$.

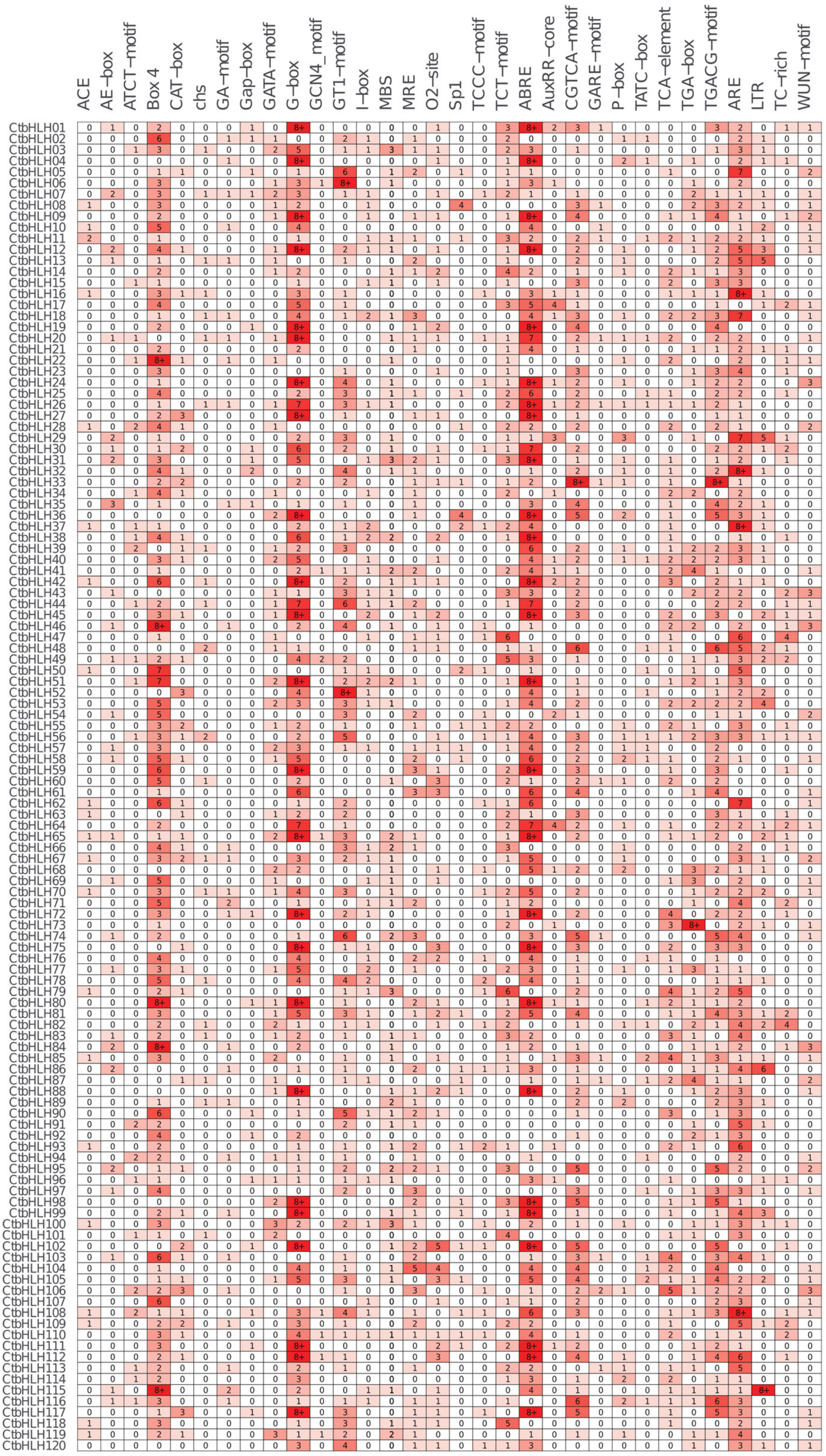

Figure S4. Cis-regulatory elements in the promoter region of *CtbHLH* genes determined from PlantCARE database. The figure represents the number of each type of regulatory element identified in the promoter sequence of *CtbHLH* genes. The darker the red color, the higher the number of regulatory elements in the promoter regions of *CtbHLH* genes.

Table S1. Information for the bHLH transcription factor family in *C.tinctorius*

Gene Locus	Gene Name	protein			Instability	Sucellular
		Length(aa)	MW(Da)	pI	Index	Location
CtAH01G0020600	*CtbHLH01*	429	47997.41	6.15	58.15	nucleus
CtAH01G0029900	*CtbHLH02*	127	14205.52	10.14	63.2	cytosol
CtAH01G0038500	*CtbHLH03*	341	38402.87	4.96	55.61	nucleus
CtAH01G0040800	*CtbHLH04*	328	35938.09	6.18	56.51	nucleus
CtAH02G0016800	*CtbHLH05*	578	65695.27	4.93	49.28	nucleus
CtAH02G0038900	*CtbHLH06*	674	73666.67	5.82	40.56	nucleus
CtAH02G0039700	*CtbHLH07*	358	39900.96	6.27	55.71	nucleus
CtAH02G0066300	*CtbHLH08*	427	47957.88	8.19	42.46	cytosol
CtAH02G0119600	*CtbHLH09*	286	30609.83	6.98	59.3	nucleus
CtAH02G0126300	*CtbHLH10*	90	10050.45	8.93	55.51	mitochondria
CtAH02G0149400	*CtbHLH11*	342	38328.59	9.18	57.75	nucleus
CtAH02G0150000	*CtbHLH12*	213	23745.1	6.5	69.52	nucleus
CtAH02G0153300	*CtbHLH13*	254	28398.47	6.11	40.41	nucleus
CtAH02G0158200	*CtbHLH14*	358	39842.52	6.01	63.32	nucleus
CtAH03G0001000	*CtbHLH15*	1465	161456.2	6.21	42.53	nucleus
CtAH03G0067400	*CtbHLH16*	338	38125.37	8.39	42.49	nucleus
CtAH03G0094800	*CtbHLH17*	336	37392.52	7.21	50.96	nucleus
CtAH03G0142200	*CtbHLH18*	240	26998.19	5.83	44.65	nucleus
CtAH03G0167300	*CtbHLH19*	452	48981.62	9.13	54.15	nucleus
CtAH03G0189300	*CtbHLH20*	224	25305.7	6.15	50.02	nucleus
CtAH03G0257900	*CtbHLH21*	306	34336.97	6.76	60.44	nucleus
CtAH03G0265000	*CtbHLH22*	452	50694.98	5.99	62.72	nucleus
CtAH03G0305900	*CtbHLH23*	261	28418.81	4.69	59.28	nucleus
CtAH04G0015500	*CtbHLH24*	294	32749.16	5.93	67.22	nucleus
CtAH04G0048700	*CtbHLH25*	273	30783.98	8.31	55.28	nucleus
CtAH04G0141400	*CtbHLH26*	198	21950.98	9.07	49.97	nucleus
CtAH04G0149100	*CtbHLH27*	249	27766.58	9.15	37.79	nucleus
CtAH04G0155900	*CtbHLH28*	413	45807.21	6.35	68.32	nucleus
CtAH04G0167000	*CtbHLH29*	397	45255.72	5.64	42.2	nucleus
CtAH04G0222200	*CtbHLH30*	375	42123.27	6.37	69.08	nucleus
CtAH04G0226500	*CtbHLH31*	269	30529.58	5.99	40.16	nucleus
CtAH05G0045700	*CtbHLH32*	252	28880.56	4.9	64.2	nucleus
CtAH05G0059100	*CtbHLH33*	698	76626.45	6.31	59.42	nucleus
CtAH05G0088900	*CtbHLH34*	283	31696.5	5.59	52.8	nucleus
CtAH05G0094600	*CtbHLH35*	260	29349.19	6.46	43.94	nucleus
CtAH05G0102400	*CtbHLH36*	337	37724	5.81	49.89	nucleus
CtAH05G0151900	*CtbHLH37*	757	85427.54	5.09	47.05	nucleus
CtAH05G0184800	*CtbHLH38*	188	20996.41	9.07	75.37	chloroplast
CtAH05G0245200	*CtbHLH39*	417	43305.13	6.25	50.51	nucleus
CtAH05G0277500	*CtbHLH40*	489	55480.57	7.09	67.24	nucleus

Table S1. *Cont.*

Gene Locus	Gene Name	protein			Instability Index	Sucellular Location
		Length(aa)	MW(Da)	pI		
CtAH06G0053200	*CtbHLH41*	375	41702.87	5.89	44.15	nucleus
CtAH06G0071400	*CtbHLH42*	513	56099.86	5.61	42.63	nucleus
CtAH06G0125600	*CtbHLH43*	586	65573.53	6.35	62.06	nucleus
CtAH06G0176400	*CtbHLH44*	420	46605.52	9	54.95	nucleus
CtAH06G0257600	*CtbHLH45*	285	31634.02	7.28	45.39	nucleus
CtAH06G0264200	*CtbHLH46*	282	30877.64	5.92	50.13	nucleus
CtAH06G0272000	*CtbHLH47*	505	54784.7	5.58	54.49	nucleus
CtAH06G0273300	*CtbHLH48*	280	31694.09	9.23	35.69	chloroplast
CtAH06G0287100	*CtbHLH49*	233	25393.33	5.28	55.85	nucleus
CtAH06G0292900	*CtbHLH50*	328	37051.15	4.89	53.36	nucleus
CtAH06G0313000	*CtbHLH51*	515	56186.75	8.07	58.65	nucleus
CtAH06G0321500	*CtbHLH52*	77	8726.93	8.66	62.91	nucleus
CtAH07G0013900	*CtbHLH53*	124	14092.12	9.46	71.65	nucleus
CtAH07G0018800	*CtbHLH54*	239	26258.5	5.64	66.31	nucleus
CtAH07G0053100	*CtbHLH55*	309	34072.28	8.39	57.54	nucleus
CtAH07G0058200	*CtbHLH56*	321	34675.18	4.93	49.59	nucleus
CtAH07G0073400	*CtbHLH57*	265	29739.53	6.36	47.94	nucleus
CtAH07G0199100	*CtbHLH58*	336	37338.69	7.72	47.03	nucleus
CtAH07G0211100	*CtbHLH59*	272	30690.71	5.72	40.18	nucleus
CtAH08G0007100	*CtbHLH60*	313	34517.06	5.5	57.21	nucleus
CtAH08G0015700	*CtbHLH61*	252	28124.63	8.86	73.21	nucleus
CtAH08G0016000	*CtbHLH62*	261	29123.84	7.1	47.31	nucleus
CtAH08G0104800	*CtbHLH63*	392	43630.32	6.09	57.66	nucleus
CtAH08G0144200	*CtbHLH64*	308	33963.01	5.86	78.17	nucleus
CtAH08G0151700	*CtbHLH65*	387	43051.17	9.29	40.52	nucleus
CtAH08G0151900	*CtbHLH66*	321	36144.41	9.4	53.83	nucleus
CtAH08G0152100	*CtbHLH67*	332	36982.17	9.07	41.03	nucleus
CtAH08G0152200	*CtbHLH68*	328	37356.38	6.03	57.46	nucleus
CtAH08G0152400	*CtbHLH69*	303	34166.63	5.73	56.74	nucleus
CtAH08G0160000	*CtbHLH70*	211	24110.44	7.78	62.23	nucleus
CtAH08G0181900	*CtbHLH71*	240	26842.88	8.65	53.91	cytosol
CtAH08G0237000	*CtbHLH72*	421	47405.97	5.2	55.6	nucleus
CtAH09G0014100	*CtbHLH73*	437	48931.58	5.99	56.83	nucleus
CtAH09G0028000	*CtbHLH74*	440	49469.61	8.7	52.82	nucleus
CtAH09G0039600	*CtbHLH75*	333	37601.28	7.02	47.48	nucleus
CtAH09G0067900	*CtbHLH76*	365	40934.77	8.61	74	nucleus
CtAH09G0072800	*CtbHLH77*	603	67139.13	5.81	58.7	nucleus
CtAH09G0091200	*CtbHLH78*	228	25637.09	6.38	59.48	nucleus
CtAH09G0091700	*CtbHLH79*	269	29795.14	7.13	58.12	nucleus
CtAH09G0096600	*CtbHLH80*	379	42457.71	5.18	51.34	nucleus
CtAH09G0102800	*CtbHLH81*	202	22396.12	4.94	54.62	chloroplast

Table S1. *Cont.*

Gene Locus	Gene Name	protein			Instability	Sucellular
		Length(aa)	MW(Da)	pI	Index	Location
CtAH09G0112700	*CtbHLH82*	312	34294.77	7.05	64.73	nucleus
CtAH09G0118900	*CtbHLH83*	290	32993.03	5.4	56.69	nucleus
CtAH09G0132000	*CtbHLH84*	217	24851.03	8.93	69.53	nucleus
CtAH09G0132200	*CtbHLH85*	503	53943.14	6.09	47.8	nucleus
CtAH09G0142600	*CtbHLH86*	191	20719.65	6.74	47.61	nucleus
CtAH09G0160300	*CtbHLH87*	167	18224.66	9.8	64.87	nucleus
CtAH09G0160500	*CtbHLH88*	242	26400.44	7.83	62.5	nucleus
CtAH09G0161900	*CtbHLH89*	91	10409.58	6.56	79.36	mitochondria
CtAH09G0162200	*CtbHLH90*	96	10707.09	9.1	77.95	nucleus
CtAH09G0174600	*CtbHLH91*	345	38457.98	6.9	61.44	nucleus
CtAH09G0192500	*CtbHLH92*	337	37534.59	4.79	60.95	nucleus
CtAH09G0233200	*CtbHLH93*	316	33550.52	6.12	51.36	nucleus
CtAH09G0235800	*CtbHLH94*	488	54381.4	6.85	51.53	nucleus
CtAH09G0248300	*CtbHLH95*	515	56316.34	5.61	66.58	nucleus
CtAH09G0257900	*CtbHLH96*	474	51930.85	6.34	51	nucleus
CtAH09G0294100	*CtbHLH97*	361	39704.52	6.31	47.77	nucleus
CtAH10G0023000	*CtbHLH98*	241	27228.02	8.86	71.7	chloroplast
CtAH10G0025700	*CtbHLH99*	768	86969.85	8.55	44.1	cytosol
CtAH10G0025800	*CtbHLH100*	295	33306.65	8.45	49.32	nucleus
CtAH10G0036500	*CtbHLH101*	312	34562.73	5.27	61.54	nucleus
CtAH10G0079000	*CtbHLH102*	547	59451.14	5.61	78.63	nucleus
CtAH10G0094800	*CtbHLH103*	605	67639.94	5.28	61.18	nucleus
CtAH10G0094900	*CtbHLH104*	622	69042.01	5	58.66	nucleus
CtAH10G0095000	*CtbHLH105*	603	67262.5	5.61	58.79	nucleus
CtAH10G0115000	*CtbHLH106*	270	30465.24	5.83	47.86	cytosol
CtAH10G0181500	*CtbHLH107*	351	38746.55	5.53	57.16	nucleus
CtAH10G0208800	*CtbHLH108*	289	32435.72	7.63	33.88	nucleus
CtAH10G0227500	*CtbHLH109*	260	28902.54	5.55	39.51	cytosol
CtAH11G0019900	*CtbHLH110*	362	41301.55	5.92	61.35	nucleus
CtAH11G0020900	*CtbHLH111*	323	35947.29	5.16	51.41	nucleus
CtAH11G0021000	*CtbHLH112*	285	31697.7	5.09	43.65	nucleus
CtAH11G0034700	*CtbHLH113*	240	26585.87	5.33	71.12	nucleus
CtAH11G0035400	*CtbHLH114*	228	25343.93	9.39	64.89	nucleus
CtAH11G0094100	*CtbHLH115*	515	58133.39	7.11	54.22	nucleus
CtAH11G0138700	*CtbHLH116*	292	31329.72	8.84	60.82	nucleus
CtAH11G0244400	*CtbHLH117*	398	44597.03	5.61	61.12	nucleus
CtAH11G0257000	*CtbHLH118*	513	56397.27	6.15	46.6	nucleus
CtAH12G0137000	*CtbHLH119*	339	35179.14	5.76	60.36	nucleus
CtAH12G0153400	*CtbHLH120*	253	29178.37	9.19	51.69	nucleus

Table S2. String interaction analysis of *CtbHLH* gene family

#node1	node2	node1_string_id	node2_string_id	coexpression	combined_score
CtbHLH04	CtbHLH92	AT1G66470.1	AT4G33880.1	0.406	0.568
CtbHLH04	CtbHLH39	AT1G66470.1	AT2G24260.1	0.181	0.405
CtbHLH04	CtbHLH89	AT1G66470.1	AT1G74500.1	0.447	0.497
CtbHLH04	CtbHLH32	AT1G66470.1	AT4G29930.3	0.171	0.408
CtbHLH04	CtbHLH05	AT1G66470.1	AT1G63650.3	0	0.63
CtbHLH05	CtbHLH92	AT1G63650.3	AT4G33880.1	0	0.548
CtbHLH08	CtbHLH31	AT1G10610.1	AT1G25330.1	0	0.457
CtbHLH08	CtbHLH114	AT1G10610.1	AT5G67060.1	0	0.61
CtbHLH08	CtbHLH78	AT1G10610.1	AT3G50330.1	0	0.61
CtbHLH10	CtbHLH57	AT1G26945.1	AT1G18400.1	0.111	0.503
CtbHLH10	CtbHLH33	AT1G26945.1	AT1G09530.2	0	0.543
CtbHLH04	CtbHLH92	AT1G66470.1	AT4G33880.1	0.406	0.568
CtbHLH10	CtbHLH48	AT1G26945.1	AT1G68920.1	0	0.587
CtbHLH100	CtbHLH41	AT3G56970.1	AT2G28160.1	0	0.977

Table S2. *Cont.*

#node1	node2	node1_string_id	node2_string_id	coexpression	combined_score
CtbHLH100	CtbHLH99	AT3G56970.1	AT3G56980.1	0.487	0.498
CtbHLH100	CtbHLH82	AT3G56970.1	AT1G22490.1	0	0.532
CtbHLH100	CtbHLH76	AT3G56970.1	AT4G00050.1	0	0.453
CtbHLH100	CtbHLH84	AT3G56970.1	AT5G50915.1	0	0.591
CtbHLH100	CtbHLH69	AT3G56970.1	AT4G37850.1	0	0.594
CtbHLH100	CtbHLH20	AT3G56970.1	AT4G14410.1	0	0.914
CtbHLH100	CtbHLH86	AT3G56970.1	AT5G54680.1	0	0.929
CtbHLH101	CtbHLH71	AT2G42300.1	AT1G68810.1	0	0.584
CtbHLH101	CtbHLH98	AT2G42300.1	AT2G41130.1	0	0.6
CtbHLH101	CtbHLH94	AT2G42300.1	AT4G16430.1	0	0.598
CtbHLH101	CtbHLH74	AT2G42300.1	AT5G51780.1	0	0.615
CtbHLH101	CtbHLH83	AT2G42300.1	AT5G65640.1	0	0.626
CtbHLH101	CtbHLH40	AT2G42300.1	AT5G56960.1	0	0.695
CtbHLH105	CtbHLH84	AT4G09820.1	AT5G50915.1	0	0.494
CtbHLH108	CtbHLH91	AT2G40200.1	AT1G69010.1	0	0.627
CtbHLH108	CtbHLH34	AT2G40200.1	AT2G14760.3	0	0.543
CtbHLH108	CtbHLH78	AT2G40200.1	AT3G50330.1	0	0.429
CtbHLH108	CtbHLH96	AT2G40200.1	AT3G07340.1	0	0.463
CtbHLH108	CtbHLH69	AT2G40200.1	AT4G37850.1	0	0.497
CtbHLH108	CtbHLH36	AT2G40200.1	AT4G34530.1	0	0.514
CtbHLH108	CtbHLH93	AT2G40200.1	AT4G02590.1	0	0.519
CtbHLH108	CtbHLH21	AT2G40200.1	AT5G46690.1	0	0.615
CtbHLH108	CtbHLH109	AT2G40200.1	AT5G51790.1	0	0.616
CtbHLH108	CtbHLH92	AT2G40200.1	AT4G33880.1	0	0.619
CtbHLH108	CtbHLH86	AT2G40200.1	AT5G54680.1	0	0.62
CtbHLH108	CtbHLH114	AT2G40200.1	AT5G67060.1	0	0.637

Table S2. *Cont.*

#node1	node2	node1_string_id	node2_string_id	coexpression	combined_score
CtbHLH21	CtbHLH95	AT5G46690.1	AT2G20180.2	0.115	0
CtbHLH21	CtbHLH76	AT5G46690.1	AT4G00050.1	0.06	0
CtbHLH21	CtbHLH33	AT5G46690.1	AT1G09530.2	0	0
CtbHLH21	CtbHLH49	AT5G46690.1	AT1G35460.1	0	0
CtbHLH21	CtbHLH88	AT5G46690.1	AT1G59640.2	0	0
CtbHLH21	CtbHLH91	AT5G46690.1	AT1G69010.1	0	0
CtbHLH21	CtbHLH34	AT5G46690.1	AT2G14760.3	0	0
CtbHLH21	CtbHLH39	AT5G46690.1	AT2G24260.1	0	0
CtbHLH21	CtbHLH97	AT5G46690.1	AT2G42280.1	0	0
CtbHLH21	CtbHLH78	AT5G46690.1	AT3G50330.1	0	0
CtbHLH21	CtbHLH94	AT5G46690.1	AT4G16430.1	0	0
CtbHLH21	CtbHLH92	AT5G46690.1	AT4G33880.1	0	0
CtbHLH21	CtbHLH36	AT5G46690.1	AT4G34530.1	0	0
CtbHLH21	CtbHLH77	AT5G46690.1	AT5G08130.5	0	0
CtbHLH21	CtbHLH54	AT5G46690.1	AT5G62610.1	0	0
CtbHLH22	CtbHLH84	AT1G32640.1	AT5G50915.1	0	0
CtbHLH24	CtbHLH88	AT3G19860.2	AT1G59640.2	0	0
CtbHLH24	CtbHLH98	AT3G19860.2	AT2G41130.1	0	0
CtbHLH24	CtbHLH54	AT3G19860.2	AT5G62610.1	0	0
CtbHLH24	CtbHLH36	AT3G19860.2	AT4G34530.1	0	0
CtbHLH26	CtbHLH96	AT4G20970.1	AT3G07340.1	0	0
CtbHLH26	CtbHLH60	AT4G20970.1	AT5G53210.1	0	0
CtbHLH29	CtbHLH71	AT2G31220.1	AT1G68810.1	0	0.115
CtbHLH29	CtbHLH37	AT2G31220.1	AT2G16910.1	0	0
CtbHLH29	CtbHLH77	AT2G31220.1	AT5G08130.5	0	0
CtbHLH29	CtbHLH40	AT2G31220.1	AT5G56960.1	0	0
CtbHLH31	CtbHLH57	AT1G25330.1	AT1G18400.1	0	0.597
CtbHLH32	CtbHLH69	AT4G29930.3	AT4G37850.1	0.047	0.49
CtbHLH32	CtbHLH36	AT4G29930.3	AT4G34530.1	0	0.115
CtbHLH32	CtbHLH92	AT4G29930.3	AT4G33880.1	0.118	0
CtbHLH32	CtbHLH82	AT4G29930.3	AT1G22490.1	0.086	0
CtbHLH32	CtbHLH39	AT4G29930.3	AT2G24260.1	0.074	0
CtbHLH32	CtbHLH34	AT4G29930.3	AT2G14760.3	0.059	0
CtbHLH32	CtbHLH91	AT4G29930.3	AT1G69010.1	0.058	0
CtbHLH32	CtbHLH78	AT4G29930.3	AT3G50330.1	0	0
CtbHLH32	CtbHLH84	AT4G29930.3	AT5G50915.1	0	0
CtbHLH33	CtbHLH95	AT1G09530.2	AT2G20180.2	0	0.501
CtbHLH33	CtbHLH38	AT1G09530.2	AT3G06120.1	0	0
CtbHLH33	CtbHLH60	AT1G09530.2	AT5G53210.1	0	0
CtbHLH34	CtbHLH71	AT2G14760.3	AT1G68810.1	0	0
CtbHLH34	CtbHLH90	AT2G14760.3	AT3G28857.1	0	0

Table S2. *Cont.*

#node1	node2	node1_string_id	node2_string_id	coexpression	combined_score
CtbHLH34	CtbHLH86	AT2G14760.3	AT5G54680.1	0	0
CtbHLH36	CtbHLH96	AT4G34530.1	AT3G07340.1	0	0.499
CtbHLH36	CtbHLH98	AT4G34530.1	AT2G41130.1	0	0
CtbHLH36	CtbHLH60	AT4G34530.1	AT5G53210.1	0	0
CtbHLH36	CtbHLH69	AT4G34530.1	AT4G37850.1	0	0
CtbHLH38	CtbHLH85	AT3G06120.1	AT3G26744.1	0.08	0.5
CtbHLH38	CtbHLH76	AT3G06120.1	AT4G00050.1	0	0
CtbHLH38	CtbHLH83	AT3G06120.1	AT5G65640.1	0	0
CtbHLH38	CtbHLH84	AT3G06120.1	AT5G50915.1	0	0
CtbHLH40	CtbHLH71	AT5G56960.1	AT1G68810.1	0	0
CtbHLH40	CtbHLH41	AT5G56960.1	AT2G28160.1	0	0
CtbHLH40	CtbHLH98	AT5G56960.1	AT2G41130.1	0	0
CtbHLH40	CtbHLH79	AT5G56960.1	AT4G36930.1	0	0
CtbHLH40	CtbHLH84	AT5G56960.1	AT5G50915.1	0	0
CtbHLH40	CtbHLH74	AT5G56960.1	AT5G51780.1	0	0
CtbHLH40	CtbHLH83	AT5G56960.1	AT5G65640.1	0	0
CtbHLH40	CtbHLH54	AT5G56960.1	AT5G62610.1	0	0
CtbHLH41	CtbHLH99	AT2G28160.1	AT3G56980.1	0	0.605
CtbHLH41	CtbHLH90	AT2G28160.1	AT3G28857.1	0	0
CtbHLH41	CtbHLH86	AT2G28160.1	AT5G54680.1	0	0
CtbHLH46	CtbHLH94	AT2G34820.1	AT4G16430.1	0	0
CtbHLH48	CtbHLH71	AT1G68920.1	AT1G68810.1	0	0
CtbHLH48	CtbHLH77	AT1G68920.1	AT5G08130.5	0	0
CtbHLH48	CtbHLH69	AT1G68920.1	AT4G37850.1	0	0
CtbHLH48	CtbHLH89	AT1G68920.1	AT1G74500.1	0	0
CtbHLH54	CtbHLH71	AT5G62610.1	AT1G68810.1	0	0
CtbHLH54	CtbHLH98	AT5G62610.1	AT2G41130.1	0	0
CtbHLH54	CtbHLH94	AT5G62610.1	AT4G16430.1	0	0
CtbHLH54	CtbHLH74	AT5G62610.1	AT5G51780.1	0	0
CtbHLH54	CtbHLH60	AT5G62610.1	AT5G53210.1	0	0
CtbHLH54	CtbHLH83	AT5G62610.1	AT5G65640.1	0	0
CtbHLH57	CtbHLH83	AT1G18400.1	AT5G65640.1	0	0
CtbHLH57	CtbHLH86	AT1G18400.1	AT5G54680.1	0	0
CtbHLH57	CtbHLH77	AT1G18400.1	AT5G08130.5	0	0
CtbHLH60	CtbHLH85	AT5G53210.1	AT3G26744.1	0.087	0.5
CtbHLH60	CtbHLH76	AT5G53210.1	AT4G00050.1	0.058	0
CtbHLH60	CtbHLH88	AT5G53210.1	AT1G59640.2	0	0
CtbHLH60	CtbHLH96	AT5G53210.1	AT3G07340.1	0	0
CtbHLH60	CtbHLH99	AT5G53210.1	AT3G56980.1	0	0
CtbHLH60	CtbHLH84	AT5G53210.1	AT5G50915.1	0	0
CtbHLH60	CtbHLH83	AT5G53210.1	AT5G65640.1	0	0

Table S2. *Cont.*

#node1	node2	node1_string_id	node2_string_id	coexpression	combined_score
CtbHLH69	CtbHLH99	AT4G37850.1	AT3G56980.1	0	0
CtbHLH69	CtbHLH93	AT4G37850.1	AT4G02590.1	0	0
CtbHLH69	CtbHLH86	AT4G37850.1	AT5G54680.1	0	0
CtbHLH71	CtbHLH84	AT1G68810.1	AT5G50915.1	0	0
CtbHLH71	CtbHLH74	AT1G68810.1	AT5G51780.1	0	0
CtbHLH71	CtbHLH91	AT1G68810.1	AT1G69010.1	0	0
CtbHLH72	CtbHLH83	AT3G24140.1	AT5G65640.1	0	0.759
CtbHLH72	CtbHLH85	AT3G24140.1	AT3G26744.1	0.087	0.5
CtbHLH74	CtbHLH84	AT5G51780.1	AT5G50915.1	0	0.115
CtbHLH74	CtbHLH89	AT5G51780.1	AT1G74500.1	0.212	0
CtbHLH74	CtbHLH82	AT5G51780.1	AT1G22490.1	0	0
CtbHLH74	CtbHLH98	AT5G51780.1	AT2G41130.1	0	0
CtbHLH74	CtbHLH83	AT5G51780.1	AT5G65640.1	0	0
CtbHLH76	CtbHLH86	AT4G00050.1	AT5G54680.1	0	0
CtbHLH77	CtbHLH91	AT5G08130.5	AT1G69010.1	0.069	0.491
CtbHLH77	CtbHLH89	AT5G08130.5	AT1G74500.1	0	0
CtbHLH77	CtbHLH94	AT5G08130.5	AT4G16430.1	0	0
CtbHLH77	CtbHLH86	AT5G08130.5	AT5G54680.1	0	0
CtbHLH78	CtbHLH79	AT3G50330.1	AT4G36930.1	0.073	0.5
CtbHLH82	CtbHLH84	AT1G22490.1	AT5G50915.1	0	0
CtbHLH83	CtbHLH98	AT5G65640.1	AT2G41130.1	0	0
CtbHLH83	CtbHLH97	AT5G65640.1	AT2G42280.1	0	0
CtbHLH83	CtbHLH84	AT5G65640.1	AT5G50915.1	0	0
CtbHLH84	CtbHLH98	AT5G50915.1	AT2G41130.1	0	0
CtbHLH84	CtbHLH85	AT5G50915.1	AT3G26744.1	0	0
CtbHLH84	CtbHLH90	AT5G50915.1	AT3G28857.1	0	0
CtbHLH84	CtbHLH99	AT5G50915.1	AT3G56980.1	0	0
CtbHLH85	CtbHLH86	AT3G26744.1	AT5G54680.1	0	0
CtbHLH86	CtbHLH91	AT5G54680.1	AT1G69010.1	0	0
CtbHLH86	CtbHLH99	AT5G54680.1	AT3G56980.1	0	0
CtbHLH86	CtbHLH93	AT5G54680.1	AT4G02590.1	0	0
CtbHLH89	CtbHLH97	AT1G74500.1	AT2G42280.1	0	0

Table S3. The FPKM values of *CtbHLHs*

Gene	White	Yellow	LightRred	DeepRred	1DAG	3DAG	5DAG	7DAG	10DAG	DAF0	DAF10	DAF20	HL_DAF10	HL_DAF20	LL_DAF10	LL_DAF20	SBS	MBS	IFS	PFS	DFS
CtbHLH01	7.86	7.76	5.61	6.33	18.16	6.39	5.52	7.91	11.42	8.74	7.72	9.02	8.79	7.63	12.85	3.81	7.08	7.39	8.13	6.54	7.11
CtbHLH02	0.43	5.47	1.10	0.81	123.68	31.19	13.8	5.82	3.33	11.83	2.07	4.96	2.11	3.1	3.38	0.06	29.99	31.85	16.31	9.27	8.14
CtbHLH03	0.04	0.19	0.1	0.05	8.8	5.82	6.68	8.72	11.02	3.94	2.77	0.51	3.3	2.2	1.09	0.39	2.16	1.52	1.6	0.56	0.95
CtbHLH04	0	0	0	0	1.45	0.52	0.29	0.21	0.02	0	0.23	0.47	0.24	0.42	0.03	0.5	0	0	0	0	0
CtbHLH05	0.05	0.41	0.14	1.95	0.09	0.45	0.73	2.81	2.03	3.19	0.3	0.29	3.52	0.54	1.93	0.08	3.89	3.2	1.75	1.26	0.62
CtbHLH06	36.81	65.37	88.88	64.49	8.98	13.02	15.84	8.54	36.63	282.18	13.63	8.92	21.14	12.04	30.88	4.58	36.77	29.85	92.82	84.63	226.45
CtbHLH07	0.05	0	0.08	0.04	51.96	35.82	48.47	6.91	9.29	94.85	25	8.16	28.13	18.25	34.87	6.97	34.99	31.42	33.43	0.04	6.65
CtbHLH08	0.01	0	0	0	1.49	7.14	4.45	2.52	0.58	3.01	0.69	0.68	0.63	0.59	0.85	0.14	2.09	1.76	1.08	0.13	0.11
CtbHLH09	4.95	9.37	8.07	54.89	28.31	17.74	14.47	26.22	23.1	50.91	5.11	26.84	6.44	7.8	9.26	6.99	153.25	324.8	70.72	14.45	19.49
CtbHLH10	0.09	0.48	5.5	0.65	0	0.02	2.01	1.45	3.79	1.01	0	0	0	0	0	0	31.12	9.16	0.52	0.55	0.04
CtbHLH11	0.04	0.03	0	0.02	16.6	0.07	0.07	0.08	0.01	0	0	4.94	0.02	0.65	0	0.04	0	0	0	0	0
CtbHLH12	0	0	0	0	0.04	0.11	0.13	0.06	0	0	1.75	0.2	0.89	0.72	0	0.12	0.02	0.05	0.03	0	0
CtbHLH13	10.28	16.07	13.9	7.32	0.02	0	0	0.26	0	0.13	0	0	0.01	0	0.03	0	2.73	4	3.4	15.74	1.56
CtbHLH14	0	0.02	0.04	0	0.32	4.36	5.02	4.1	4.6	1.01	2.25	0.26	1.64	1.44	1.06	0.89	2.24	1.7	0.45	0.08	0.04
CtbHLH15	2.43	2.79	1.82	3.54	7.2	2.6	3.04	3.34	14.11	7.27	1.98	3.03	4.72	2.96	3.25	1.65	4.5	4.33	4.83	4.62	3.51
CtbHLH16	0.33	0.05	0.19	0.19	0.13	6.44	27.5	22.03	21.87	2.79	0	0	0	0	0	0	2.9	2	1.62	1.17	0.51
CtbHLH17	0.50	0.51	0.61	0.32	0.34	0.89	1.36	1.1	0.96	0.38	0.06	0.16	0.09	0.2	0.13	0.02	0.11	0.09	0.29	1.04	0.23
CtbHLH18	0.00	0.00	0.00	0.00	0	0	0	0	0	0.02	0.01	0.03	0.09	0.05	0.12	0	0.09	0.02	0	0.02	0
CtbHLH19	53.19	46.94	34.31	27.34	0.05	1.04	7.68	6.24	26.58	0.06	0.34	0.69	0.38	0.46	0.28	0.25	0.69	2.55	9.84	36.29	1.85
CtbHLH20	13.51	14.69	13.82	13.81	23.53	17.8	17.39	21.6	25.52	24.03	4.43	2.29	7.75	4.7	12.83	2.3	18.69	21.73	19.38	16.24	23.56
CtbHLH21	0.02	0	0.02	0	0.01	3.46	4.34	1.77	0.9	1.49	0.88	0.01	2.89	0.13	3.29	0.04	8.27	2.76	1.63	0.02	0.02
CtbHLH22	0.64	1.44	2.69	0.53	0.76	0.04	0.1	0.09	0.04	79.96	0.1	1.5	0.45	0.16	3.98	0.02	34.93	14.45	29.56	8.42	162.4
CtbHLH23	43.11	66.67	73.67	112.47	118.28	33.64	22.29	37.23	49.26	28.55	18.27	42.05	26.96	32.26	37.32	21.17	40.3	31.16	37.56	60.24	132.7

Table S3. *Cont.*

Gene	White	Yellow	LightRred	DeepRred	1DAG	3DAG	5DAG	7DAG	10DAG	DAF0	DAF10	DAF20	HL_DAF10	HL_DAF20	LL_DAF10	LL_DAF20	SBS	MBS	IFS	PFS	DFS
CtbHLH24	12.1	11	6.03	16.01	10.54	4.67	4.75	3.39	8.94	5.64	2.5	4.98	2.74	2.58	3.12	3.7	5.03	3.65	4.2	5.2	13.17
CtbHLH25	0.55	0.53	0.13	0.22	2.37	8.07	8.44	7.39	11.08	3.48	4.16	4.91	5.15	4.67	1.7	1.59	3.32	2.02	2.14	1.97	1.96
CtbHLH26	0.04	0.04	0	0	0.02	8.87	24.61	4.18	16.23	0	0.03	0	0.04	0	0.19	0	0.78	0.03	0.06	0.16	0
CtbHLH27	0.71	1.59	2.74	1.91	0.16	1.77	3.53	4.93	12.12	0.44	0.25	2.32	0.44	1.76	0.57	1.09	0.7	0.26	0.48	1.67	0.23
CtbHLH28	20.88	17.12	30.33	14.75	83.54	9.88	9.69	18.49	25.24	1.15	1.44	16.21	1.08	6.65	0.32	1.52	1.29	1.26	3.6	8.64	18.2
CtbHLH29	0.82	0.5	0.34	0.25	0.18	0.52	1.4	0.57	1.78	0.53	0.23	0.3	0.45	0.52	0.91	0.24	0.59	0.6	0.32	0.08	0.02
CtbHLH30	0.47	0.37	0.76	0.48	2.23	3.86	4.44	6.17	2.56	1.56	2.92	0.52	2.91	1.64	2.56	2.01	2.54	0.65	0.54	0.63	0.22
CtbHLH31	10.85	21.36	24.08	6.18	0	0	0	0	0	5.3	0	0.01	0	0	0.01	0	4	5.94	12.66	20.78	0.88
CtbHLH32	4.56	3.32	0.59	0.73	1.3	4.81	5.95	3.88	10.24	1.64	0.08	0.11	0.14	0.07	1.21	0.24	3.45	2.42	1.27	2.15	0.98
CtbHLH33	3.34	3.56	3.24	1.77	18.66	15.15	18.55	10.48	23.76	21.36	1.95	2.51	3.9	2.2	4.88	1.59	25.13	32.08	18.27	5.92	23.56
CtbHLH34	1.97	2.29	2.01	2.58	1.67	3.2	3.1	3	9.04	4.45	1.86	2.63	3.16	2.51	2.85	2.08	3.65	3.61	1.99	2.19	2.17
CtbHLH35	0	0	0	0	1.73	0	0	0.02	0	0.51	0.33	0.03	0.59	0.05	2.64	0.07	0.2	0.23	0.24	0.05	0
CtbHLH36	6.19	5.04	15.03	6.72	10.5	14.47	22.03	18.56	57.08	19.19	0.31	0.18	0.33	0.08	1.09	0.12	7.14	14.9	21.23	9.98	38.27
CtbHLH37	0	0.01	0.01	0	0.01	0.01	0	0	0	0	0.01	0.03	0.01	0.01	0	0.01	6.93	0	0	0	0.07
CtbHLH38	0	0.01	0.01	0	0.26	3.01	2.95	0.02	0	0.05	0.31	0.14	0.11	0.29	0.22	0.07	0.07	0	0	0	0
CtbHLH39	0.64	3.47	0.51	1.3	0.47	3.21	3.75	9.01	2.1	2.69	1.32	0.47	22.38	9.44	44.41	5.26	6.27	3.72	2.65	2.74	4.46
CtbHLH40	0	0.02	0	0	0	0	0	0	0.06	0	0	0	0	0	0	0	0	0	0	0.01	0.07
CtbHLH41	2.01	0	1.69	0.33	0.46	0.65	3.06	4.36	1.17	0.59	0.27	1.69	0.14	0.12	0.1	0.1	0.63	0.68	1.09	1.57	0.2
CtbHLH42	111.02	81.65	80.39	50.09	10.05	15.39	21.57	16.06	61.83	58.52	1.02	1.37	3.62	2.48	5.91	1.1	22.49	25.62	83.19	113.93	38.81
CtbHLH43	7.31	10.59	22.47	13.36	17.62	2.11	2.09	5.25	4.35	1.81	0.15	0.39	0.68	0.41	0.84	0.05	0.3	0.3	0.91	2.18	29.52
CtbHLH44	3.82	3.49	2.03	0.62	1.3	0.3	9.02	1.05	16.28	1.05	0.31	8.72	0.26	3.77	0.04	1.15	0.29	0.83	1.04	2.2	8.87
CtbHLH45	0	0.11	0.08	0.05	18.54	2.39	2.61	1.44	0.56	0.01	0.28	0.33	0.32	0.28	0.26	0.08	0.5	0.01	0.02	0	0.07
CtbHLH46	0	0	0	0	0	0.01	0	0.09	0	0.06	0	0	0	0.01	0	0	0.21	0.02	0.02	0	0.05

Table S3. *Cont.*

Gene	White	Yellow	LightRred	DeepRred	1DAG	3DAG	5DAG	7DAG	10DAG	DAF0	DAF10	DAF20	HL_DAF10	HL_DAF20	LL_DAF10	LL_DAF20	SBS	MBS	IFS	PFS	DFS
CtbHLH47	6.2	4.47	4.07	7.94	8.32	10.84	8.08	1.41	3.13	45.9	35.98	17.6	55.79	31.91	66.65	12.23	29.57	24.7	20.49	5.91	10.47
CtbHLH48	0.45	0.35	0.12	0.59	1.86	3.1	3.27	2.39	2.08	2.51	0.06	0.14	0.16	0.14	0.3	0.15	1.23	1.16	1.12	0.49	0.21
CtbHLH49	3.71	7.21	5.25	9.53	41.74	4.28	4.91	4.65	13.08	7.64	3.77	60.47	3.99	8.03	3.85	7.7	6.6	6.74	8.76	9.93	19.27
CtbHLH50	0	0	0	0	0.27	0.5	0.48	0.85	0.15	0	0	0	0	0	0.06	0.01	0	0	0	0	0
CtbHLH51	36.37	31.16	36.88	15.2	14.35	5.4	9	8.41	31	33.01	2.01	2.01	4.32	3.08	5.69	2.91	17.42	23.98	21.65	14.3	12.05
CtbHLH52	0	0	0	0	0.1	0	0	0.06	0.02	2.13	0.04	0	0.06	0.03	0	0	0.22	0.2	5.17	0	0
CtbHLH53	7.66	3.93	2.39	0.28	0.02	0	0.02	0.02	0	0.06	0	0	0	0	0	0	0.15	0.29	1.55	0.56	0
CtbHLH54	86.07	52.72	84.45	79.96	3.44	4.63	2.92	0.93	1.03	20.16	1.08	2.51	1.85	1.11	5.03	1.39	61.26	143.22	43.71	56.27	10.31
CtbHLH55	0.02	0.02	0	0	0.2	0.37	0.62	1.28	0.4	0.25	0.14	0.01	0.25	0.16	0.08	0.08	0.47	0.71	0.18	0.16	0
CtbHLH56	15.04	18.15	18.66	18.46	17.59	8.91	9.28	10.29	13.42	17.77	6.46	13.04	10.91	9.72	17.06	7.55	21.16	22.33	20.83	25.08	27.63
CtbHLH57	0.15	0.29	1.07	0	0.61	2.65	7.72	13.13	17.35	0.05	0	0	0	0	0	0	0.01	0.02	0	0	0.51
CtbHLH58	0.7	0.92	0.82	0.4	0.66	1.58	1.26	8.59	5.19	4.66	0.81	0.18	1.01	0.68	0.71	0.23	1.91	2.61	2.85	3.45	1.77
CtbHLH59	0	0	0	0	0.17	0.01	0	0.03	0.35	0.06	0.03	0	0.1	0.1	1.39	0.1	0.02	0	0	0	0.84
CtbHLH60	0	0	0	0	16.68	3.29	1.66	0.1	0	0.05	1.03	0.63	0.96	1.27	1.5	0.71	0.02	0.01	0.01	0.03	0
CtbHLH61	0.14	0.27	2.78	7.6	5.32	18.3	21.79	18.45	67.24	5.75	12.42	9.53	14.48	11.47	3.64	1.47	6.92	5.23	32.12	84.18	31.16
CtbHLH62	0.24	0.28	0	0	0.04	2.9	3.94	3.21	10.28	0	0	0	0	0	0	0	0	0.05	0	0.01	0
CtbHLH63	5.25	7.44	4.37	5.11	12.44	1.96	6.21	3.32	10.1	6.46	0.73	1.12	9.92	4.05	27.16	1.78	5.5	5.57	5.93	6.78	5.75
CtbHLH64	0.06	0.02	0.04	0	0.13	1.05	1.11	9.53	1.12	2.19	1.68	0.11	1.87	1.17	1.24	0.35	8.67	1.36	1.05	0.47	0.54
CtbHLH65	0	0	0	0	0.78	0.25	0.25	2.7	0.25	0.01	0.29	0.32	0.18	0.45	0.38	0.03	0	0	0.02	0	0
CtbHLH66	0	0	0	0	0.33	0.27	0.33	2.05	0.59	0.01	0.13	0.16	0.04	0.14	0.09	0.07	0.24	0.15	0	0	0
CtbHLH67	0	0	0	0	0.44	0.55	0.6	5.76	0.9	0	0.46	0.25	0.31	0.47	0.23	0.05	0	0	0	0	0
CtbHLH68	2.96	6.93	7.02	7.01	1.82	6.22	7.35	0.68	0.96	27	0.96	1.9	0.84	1.11	1.67	0.45	5.63	5.91	10.61	8.63	5.41
CtbHLH69	13.24	14.74	25.96	33.48	11.47	5.95	8.32	7.88	6.28	16.56	5.01	6.68	5.13	5.86	6.36	5.35	6.16	6.75	7.63	14.65	37.96

Table S3. *Cont.*

Gene	White	Yellow	LightRred	DeepRred	1DAG	3DAG	5DAG	7DAG	10DAG	DAF0	DAF10	DAF20	HL_DAF10	HL_DAF20	LL_DAF10	LL_DAF20	SBS	MBS	IFS	PFS	DFS
CtbHLH70	30.44	41.44	37.32	7.92	0.11	0.39	0.58	0.34	0.13	28.59	0.22	0.11	0.27	0.58	1.45	0.44	8.53	7.19	18.64	8.58	2.7
CtbHLH71	0	0	0	0	0.03	1.74	1.79	3.7	2.56	0.78	1.1	0.03	0.69	0.4	0.91	0.23	0.66	0.13	0.02	0	0
CtbHLH72	0	0	0	0	0.35	1.46	2.71	0.12	0.05	0.03	0	0.05	0.02	0.02	0	0	0.05	0.01	0	0.05	0.07
CtbHLH73	0.07	0.39	0.27	0.08	0.35	2.93	6.63	1.13	4.29	0	0.03	0.02	0.06	0.08	0.13	0.02	0.71	0.22	0.12	0.18	0.05
CtbHLH74	0.12	0.06	0.02	0.02	0	1.36	1.41	1.7	1.51	0.01	0.03	0.02	0.09	0.07	0.08	0.06	0.14	0.05	0.04	0.09	0.1
CtbHLH75	1.52	16.77	3.09	0.82	5.14	9.68	9.76	11.16	46.27	1.01	0.15	0.12	0.33	0.34	0.64	0.09	1.71	1.08	7.9	19.43	2.58
CtbHLH76	1.69	2.05	3.53	4.75	0.92	2.8	17.74	6.31	43.31	0.52	0.98	1.13	0.31	0.73	0.08	0.36	0.13	0.06	0.55	1.19	0.57
CtbHLH77	40.12	28.15	17.77	22.02	10.85	7.88	6.52	5.71	6.86	22.72	0.81	0.59	1.86	0.95	3.77	0.89	6.11	5.61	10.73	10.03	5.38
CtbHLH78	0	0	0	0	59.85	185.07	69.22	8.82	19.46	0	0.19	1.66	0.44	0.76	0.08	0.09	0.03	0	0	0	0
CtbHLH79	6.18	10.13	5.83	7.12	55.98	29.14	27.25	1.53	0.4	1.91	7.57	7.9	8.23	8.35	8.98	4.68	40.61	22.78	5.22	10.28	2.67
CtbHLH80	0	0	0	0.03	0	0.89	0.71	0.16	0.08	0	0	0	0	0	0	0	0	0	0	0	0
CtbHLH81	0.11	0.22	0.15	0.57	5.04	27.63	22.53	7.78	9.05	0.82	5.85	57.54	4.4	21.58	1.23	9.58	2.53	1.6	0.76	0.47	0.35
CtbHLH82	0	0	0.04	0.06	2.28	2.96	2.72	3.23	9	1.25	2.09	0.84	1.72	2.28	2.55	0.88	1.66	0.9	0.54	0.14	1.15
CtbHLH83	0.04	0.14	0.47	0.78	8.95	8.75	1.73	19.65	0.02	20.78	19.22	0.77	30.43	8.28	27.33	2.2	10.01	6.96	8.31	1.92	0.86
CtbHLH84	0	0	0	0	1.61	8.55	3.45	0.15	0.06	0.2	0	0.02	0	0.06	1.39	0.11	0.11	0	0	0	0.04
CtbHLH85	1.81	1.57	2.43	0.93	25.82	34.11	18.7	7.15	7.21	62.33	81.59	19.55	106.29	66.53	56.78	7.79	47.7	33.64	25.48	3.6	5.1
CtbHLH86	59.34	65.01	64.49	75.21	42.18	53.25	62.77	54.99	144.98	81.37	32.03	29.93	47.37	31.36	76.98	17.68	110.1	109.94	109.18	97.73	124.54
CtbHLH87	0.38	0.23	0.33	0.86	0.81	0.22	0.38	0.33	0.18	0.59	0.02	0.25	0.12	0.14	0.09	0.26	0	0.02	0	0.1	0.18
CtbHLH88	82.75	92.39	107.15	172.82	11.48	5.21	3.99	4.2	8.22	33.27	2.43	5.76	4.86	6.17	9.25	2.37	20.48	43.62	88.19	112.09	47.58
CtbHLH89	0	0	0	0	28.81	3.17	2.83	10.24	1.98	0.07	1.55	1.78	0.52	1.99	1.34	2.38	0.03	0	0	0.02	0.19
CtbHLH90	15.91	14.35	43.41	13.64	0.07	0.07	0.24	0.16	0.12	0.11	0.02	0.02	0.02	0.01	0.05	0.02	0.03	0.05	3.01	8.32	0.07
CtbHLH91	2.51	3.07	2.68	5.78	66.93	6.17	7.95	5.42	15.72	4.56	1.27	3.76	2.96	2.5	8.1	2.59	4.57	4.2	3.97	3.23	4.24
CtbHLH92	0	0	0	0	0.03	0.56	0.43	0.13	0.06	0	0	0	0	0.01	0	0	0	0	0	0	0

Table S3. *Cont.*

Gene	White	Yellow	LightRred	DeepRred	1DAG	3DAG	5DAG	7DAG	10DAG	DAF0	DAF10	DAF20	HL_DAF10	HL_DAF20	LL_DAF10	LL_DAF20	SBS	MBS	IFS	PFS	DFS
CtbHLH93	4.18	9.39	5.58	11.52	10.29	9.98	12.09	22.66	31.96	12.83	5.61	14.49	12.72	8.5	12.66	4.53	13.9	10.11	9.82	13.92	15.67
CtbHLH94	40.17	45.77	43.46	42.02	23.34	10.3	8.15	8.94	11.97	24.95	9.94	42.03	16.43	31.94	17.91	22.42	22.11	32.59	30.27	31.14	38.51
CtbHLH95	1.02	0.68	1.36	0.53	6.41	11.71	13.84	7.28	27.31	1.34	10.68	11.83	8.64	11.12	0.59	8.87	5.82	4.43	0.88	0.7	0.89
CtbHLH96	3.33	0.35	1.14	0.45	39.47	4.42	3.3	3.09	2.61	9	2.75	3.38	5.66	3.46	2.58	1.22	4.05	3.08	5.47	0.41	0.94
CtbHLH97	0.55	1.5	1.42	1.07	54.88	17	18.04	10.18	23.38	0.97	7.16	21.54	7.38	12.46	1.67	6.88	0.69	0.77	1.36	1.91	2.83
CtbHLH98	0.03	0.03	0	0	0.45	0.88	0.93	1.61	1.14	0.26	0.71	1.49	0.51	0.56	0.73	0.65	1.37	0.74	0.51	0.12	0.04
CtbHLH99	2.03	4.19	1.71	7.37	0.01	0.24	0.29	0.28	1.32	0.05	0.03	0.01	0.04	0.01	0.05	0.03	0.14	0.02	0.7	2.64	4.64
CtbHLH100	0	0.02	0.15	0.1	22.6	4.45	3.1	1.13	22.45	26.2	5.92	20.05	7	15.6	0.01	0.02	8.48	8.51	6	4.3	6.03
CtbHLH101	25.73	15.79	27.31	26.13	48.77	10.23	18.76	9.28	34.72	36.88	7.8	21.65	10.19	9.96	12.76	6.1	34.69	31.68	20.73	19.83	23.4
CtbHLH102	7.46	6.14	6.31	3.42	25.31	8.51	6.79	1.97	8.63	3.07	0.52	10.56	0.37	1.27	0.15	3.25	10.36	7.23	5.56	3.92	2.01
CtbHLH103	0.42	2.35	0.04	0.12	0.91	0.64	0.23	0.28	0.05	6.69	0.64	1.33	4.28	1.56	2.49	0.13	5.55	4.65	3.7	3.7	1.84
CtbHLH104	0.04	3.27	0.04	0.04	1.13	0.96	0.86	0.06	0.14	6.19	1.02	1.83	2.02	2.4	1.32	0.62	11.17	10.19	8	6.53	7.12
CtbHLH105	6.09	4.7	0.73	0.54	1.99	5.92	5.89	0.27	1.13	3.41	0.06	0.54	1.78	1.03	3.53	0.35	2.96	2.23	2.52	3.68	3.17
CtbHLH106	0.25	0.14	0.13	0.23	3.29	1.29	1.8	0.81	0.24	3.75	0.85	0.9	0.77	1.24	2.36	0.2	2.58	2.11	1.72	0.3	0.66
CtbHLH107	0.75	1.07	6.05	7.13	4.16	1.87	1.72	6.52	0.28	31.06	11.18	6.54	17.08	15.43	9.94	2.76	45.73	27.22	36.26	3.99	20.39
CtbHLH108	0.03	0.11	0	0	1.3	0.14	0.4	0.28	3.14	0.09	0.01	0.08	0	0	0	0	0.04	0.02	0.02	0.04	0.34
CtbHLH109	0	0	0	0	0	0.32	0.84	4.22	2.1	0.07	0	0	0	0	0.02	0	0	0	0.02	0.07	0.01
CtbHLH110	0.51	0.86	0.53	0.34	19	1.68	1.65	10.25	9.56	1.14	1.26	1.99	0.27	0.8	0.13	1.26	1.81	0.61	0.53	0.11	12.21
CtbHLH111	0	0	0.03	0	0	0	0.11	0.95	0.09	0.14	0	0.01	0	0	0	0	0	0	0	0	0
CtbHLH112	0	0	0	0	0	0.11	0.26	0.25	1.84	0	0	0	0	0	0	0	0.14	0.11	0	0	0
CtbHLH113	4.87	12.76	15.6	8.64	0.76	0.99	0.95	2.42	0.62	7.9	0.04	0	0.23	0.63	0.25	0.04	28.97	16.98	9.46	9.89	1.73
CtbHLH114	0	0	0	0.04	33.31	34.43	18.54	0.45	0.75	0.03	1.36	1.37	0.76	1.52	0.29	0.22	0.07	0	0	0.1	0.41
CtbHLH115	21.9	21.19	20.3	17.69	33.87	14.21	11.21	11.73	18.13	60.24	9.14	11.74	12.05	12.92	20.53	6.99	31.56	37.9	28.43	17.09	30.74

Table S3. *Cont.*

Gene	White	Yellow	LightRred	DeepRred	1DAG	3DAG	5DAG	7DAG	10DAG	DAF0	DAF10	DAF20	HL_DAF10	HL_DAF20	LL_DAF10	LL_DAF20	SBS	MBS	IFS	PFS	DFS
CtbHLH116	0.75	0.41	1.55	0.81	27.13	3.65	3.32	4.39	0.94	5.27	0.21	0	10.56	3.56	22.17	2.32	0.63	0.33	1.78	0.94	17.86
CtbHLH117	11.88	11.83	7.32	5.49	10.87	14.22	19.52	16.94	29.79	5.13	2.29	2.24	8.16	4.78	11.07	2.76	3.5	5.99	7.48	9.08	4.44
CtbHLH118	34.86	21.64	32.43	22	2.04	10.07	15.31	9.36	11.69	13.2	0.93	0.98	2.43	1.5	5.53	0.75	7.38	4.79	17.12	22	5.71
CtbHLH119	41.05	37.2	13.1	16.02	1.35	2.72	3.06	9.06	1.86	1.56	3.31	1.5	5.12	2.88	10.66	1.21	7.12	4.73	3.45	6.95	2.63
CtbHLH120	9.36	16.16	11.44	10.12	8.32	6.63	7.24	12.38	10.46	1.96	1.99	4.82	2.49	3.91	2.72	2.32	6.32	4.8	6.18	12.69	9.66

Table S4. Primers used in the quantitative real-time PCR analysis

Gene Name	Forward primer sequence	Reverse primer sequence
CtbHLH03	CAAACCCCAGTTGTTTTCAC	TCTTCGTCTTTCCGCCAT
CtbHLH06	CACCTACCACCACCGTACCT	TAACCGTCTCCCCAACCTAA
CtbHLH12	CAAGCCAATCCCACAGAAAG	TGATGTAGCAGCAACTCCCA
CtbHLH20	ATGTCGCATCCACCGATC	TCTCCCTCCTTTGCCTCTC
CtbHLH24	CACCCCCCTTCGTATCCCTA	CTTCCGCTCTCCCCTTGTTC
CtbHLH26	GCTCCATTCTCTTGTTCCTCA	CAAACTATCCTTTTCTTCCGTC
CtbHLH42	CAACACATCATCCTACAATACTCC	TGCTGCCCTCTCCACAA
CtbHLH51	AACGGGTAGGGAACGACGT	TTTGGGTTTGATTGAGGCA
CtbHLH70	AATGCCTCATCACCTCCTTC	CTTCTTGGGGTTCACTTCTACA
CtbHLH74	CCGATGGGGTTGAAGATG	TCGATTCGAGACCAGGAGC
CtbHLH88	AACAGTCAGACAAACCCTCCA	GAACCCAAGATTACCACAAAAG
CtbHLH99	GTGAGCGTTTTAGGCGAGA	ACAAAGGAGGGGGGGAT
CtbHLH102	GCAGAGCCAAAACCAGAGA	GTAATGAAGCCAAGCCGAC
CtbHLH105	GCCCATTCTTCCAACACC	CTCAAAGTCCGACCCGTT
CtbHLH119	AGGTGGTGAAGCTGATGGA	AGATGGCAGTGGCAAGTGA
Ct60S	TGGAGCTCATCAAGAAGGG	GGTAAGGACCACAAGACCGTA
